# Mechanism-based target therapy in primary biliary cholangitis: opportunities before liver cirrhosis?

**DOI:** 10.3389/fimmu.2023.1184252

**Published:** 2023-05-30

**Authors:** Yushu Yang, XiaoSong He, Manuel Rojas, Patrick S. C. Leung, Lixia Gao

**Affiliations:** ^1^ Department of Rheumatology and Immunology, The Second Hospital of Hebei Medical University, Shijiazhuang, Hebei, China; ^2^ Division of Rheumatology, Allergy, and Clinical Immunology, University of California, Davis, Davis, CA, United States; ^3^ Center for Autoimmune Diseases Research (CREA), School of Medicine and Health Sciences, Universidad del Rosario, Bogota, Colombia

**Keywords:** primary biliary cholangitis, immune cells, bile acids, nuclear receptors, liver fibrosis

## Abstract

Primary biliary cholangitis (PBC) is an immune-mediated liver disease characterized by cholestasis, biliary injuries, liver fibrosis, and chronic non-suppurative cholangitis. The pathogenesis of PBC is multifactorial and involves immune dysregulation, abnormal bile metabolism, and progressive fibrosis, ultimately leading to cirrhosis and liver failure. Ursodeoxycholic acid (UDCA) and obeticholic acid (OCA) are currently used as first- and second-line treatments, respectively. However, many patients do not respond adequately to UDCA, and the long-term effects of these drugs are limited. Recent research has advanced our understanding the mechanisms of pathogenesis in PBC and greatly facilitated development of novel drugs to target mechanistic checkpoints. Animal studies and clinical trials of pipeline drugs have yielded promising results in slowing disease progression. Targeting immune mediated pathogenesis and anti-inflammatory therapies are focused on the early stage, while anti-cholestatic and anti-fibrotic therapies are emphasized in the late stage of disease, which is characterized by fibrosis and cirrhosis development. Nonetheless, it is worth noting that currently, there exists a dearth of therapeutic options that can effectively impede the progression of the disease to its terminal stages. Hence, there is an urgent need for further research aimed at investigating the underlying pathophysiology mechanisms with potential therapeutic effects. This review highlights our current knowledge of the underlying immunological and cellular mechanisms of pathogenesis in PBC. Further, we also address current mechanism-based target therapies for PBC and potential therapeutic strategies to improve the efficacy of existing treatments.

## 1 Introduction

Primary biliary cholangitis (PBC) is a chronic and progressive autoimmune cholestatic liver disease, which generally develop to cirrhosis and liver failure after 10–20 years without treatment. The global prevalence of PBC is estimated at 14.6 per 100 000 population, ranging from 1.91 to 40.2 (1). Both the incidence and prevalence of this condition is increasing, with the Asia-Pacific, Europe, and North America reporting annual incidences of 0.84, 1.86, and 2.75 per 100,000 population, respectively (2). The etiology and pathogenesis of PBC remain unclear, and the clinical course of the disease is insidious and heterogeneous, with variable individual responses to drug therapy. Biliary injury is a consequence of dysregulated intrahepatic and systemic immune responses, which result in cholestasis and eventual development of liver cirrhosis. The primary objective of PBC treatment is to prevent disease progression and the development of cirrhosis and liver failure. Collagen is a major extracellular matrix in fibrotic tissues (3), and its synthesis increases in PBC. The metabolic regulation of collagen biosynthesis and degradation (4) may counteract with the increased synthesis in the early stages of PBC, but cannot compensate for the extensive collagen synthesis at the late stages of PBC, resulting in gradual development of liver cirrhosis (5). Therefore, the development of new therapies for PBC requires two distinct approaches. In the early stages of the disease, the primary focus is on regulating the immune response, controlling inflammation, and improving metabolism. In the later stages, the emphasis shifts towards controlling collagen synthesis and increasing collagen degradation. Agents targeting immune-mediated pathogenesis and anti-inflammatory are probably most effective in the early stage of PBC, while anti-cholestatic and anti-fibrotic therapies are emphasized in the late stage. Although ursodeoxycholic acid (UDCA) and obeticholic acid (OCA) are approved by the Food and Drug Administration (FDA) as first and second line of therapy respectively, cirrhotic patients hardly benefit and some PBC patients are non-responsive (6, 7). This review summarizes the advances in the research of PBC pathogenesis and related treatment, with a perspective on the window of opportunity in slowing the disease progression and prevent the development of fibrosis and cirrhosis.

## 2 Novel advances targeting immune factors

**Figure 1:**
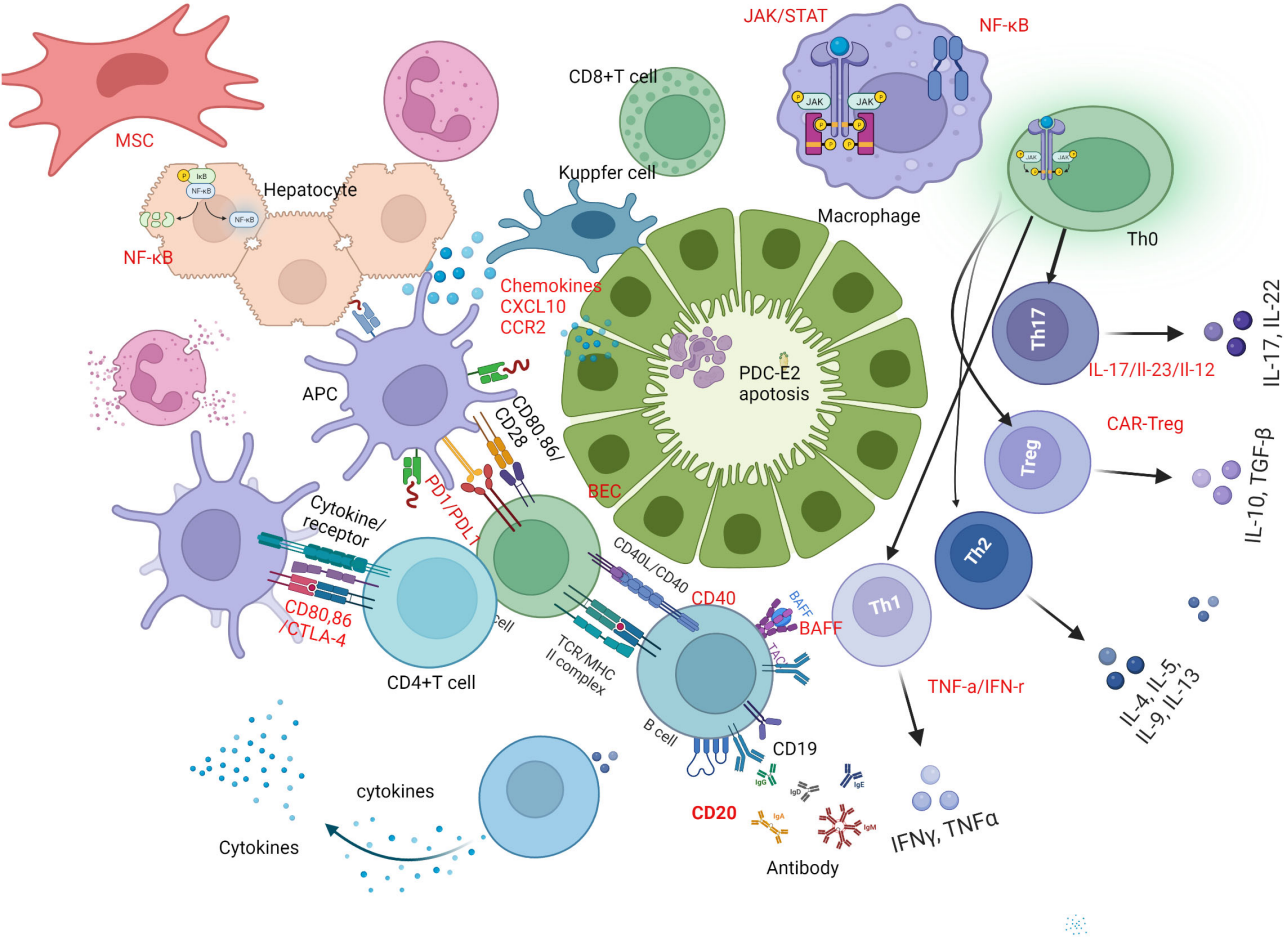
Immune mechanism based therapeutic strategies in PBC. B-cell activating factor of the tumor necrosis factor family (BAFF) and CD20-targeted therapy play a crucial role in breaking immune tolerance and stimulating immune responses in primary biliary cholangitis (PBC). Promising novel therapeutic targets for PBC treatment are highlighted in red. One potential strategy is B-cell targeted therapy, including the use of anti-CD20, anti-BAFF, or a combination of both. Another approach is T-cell-directed immunotherapy, which involves inhibiting Th1 and Th17 cell differentiation by regulating related cytokines, up-regulating Treg function and number via chimeric antigen receptor-modified Tregs (CAR-Tregs). Additionally, interfering with costimulatory signals between cells, such as targeting CTLA-4, PD-1, and CD40, has shown potential in treating PBC. Regulating related cytokines, targeting chemokines, and inhibiting signal pathways involved in PBC pathogenesis, such as monoclonal antibodies against CXCL10, JAK inhibitors, or inhibitors of the NF-κB signal pathway, represent a fourth potential approach. Finally, mesenchymal stem cells (MSCs) can be used to regulate innate and adaptive immune responses by differentiating induced pluripotent stem cells. BEC, biliary epithelial cell; PBC, primary biliary cholangitis; BAFF, B-cell -activating factor; Th1, type 1 T helper cell; Th17, type 17 T helper cell; Treg, regulatory T cell; CAR, chimeric antigen receptor; CTLA-4,cytotoxic T-lymphocyte–antigen-4; PD-1, Programmed death-1; CXCL10, chemokine (C-X-C motif) ligand 10; MSC, mesenchymal stem cells.

Innate and adaptive immunity are vigorously involved at different stages of PBC. Innate immune cells include monocytes and macrophages, dendritic cells (DCs), and natural killer (NK)/natural killer T (NKT) cells are active players in the early stage of PBC (8, 9). Adaptive immune cells including antibody secreting B cells and CD3+ and CD4+ or CD8+ lymphocytes, are also critical in the early stages of the disease whereas CD8+T cells are predominant around the damaged interlobular bile ducts in early stage of PBC (10). Increasing evidence confirms the participation of different T cell subpopulations in PBC pathogenesis, including Th1, Th17, regulatory T cells (Tregs), follicular helper T (Tfh) cells, and follicular regulatory T (Tfr) cells (11). Consequently, treatment targeting immune cells and cytokines profiles have drawn much attention (**Figure 1**).

### 2.1 Targeting immune cells and related cytokines

#### 2.1.1 Targeting B cells and related cytokines

The presence of antimitochondrial antibodies (AMA) is considered the serological hallmark of PBC. The disease specificity of AMA and high levels of serum immunoglobulin (Ig) M signifies the involvement of B cells mediated mechanisms in PBC (12, 13). Compared to healthy individuals, the frequency of CD19^+^ B cells are highly increased in livers of PBC patients, resulting in production of higher amounts of interleukin (IL)-6, IL-10, interferon (IFN)-γ and tumor necrosis factor (TNF)-α. This can be attributed to the functional abnormality of CD19^+^CD24^hi^CD38^hi^ B regulatory cells, increase in CD3^+^CD4^+^CXCR5^+^ICOS^+^ Tfh and CD38^+^ plasma cells in the peripheral blood, and elevation of serum IL-21 in PBC patients in comparison to healthy controls (14). These changes positively correlated with the levels of Ig and autoantibodies in this condition (15). In patients with PBC, circulating CD19^+^ B cells are reduced after treatment with UDCA (16).

Targeting B cell is a logical treatment strategy in PBC. Rituximab is an anti-CD20 monoclonal antibody that selectively depletes B cells. In animal studies, although anti-CD20 and anti-CD79 antibodies successfully depleted B cells and reduced the autoantibody production, it also elevated liver enzymes and aggravated PBC-like liver lesion (17). In PBC patients with incomplete response to UDCA, rituximab treatment could improve alkaline phosphatase (ALP) levels, reduce serum AMA titer, increase Treg cells numbers, and modulate cytokine production (18, 19). A phase-2 randomized controlled trial demonstrated that rituximab was safe, but did not improve symptoms of the fatigue (20). Subsequent clinical trials using a chimeric antibody against human CD20 (hCD20) showed limited efficacy. Furthermore, another humanized anti-human CD20 antibody (TKM-011) treatment, also impaired autoimmune cholangitis compared with rituximab in a mouse model of PBC (21). Hence, the efficacy of monotherapy using anti-CD20 in the treatment of PBC remains uncertain.

B-cell -activating factor (BAFF) belonging to the TNF family and a proliferation inducing ligand were thought to be involved the pathogenesis of PBC. Serum levels of BAFF are increased in PBC patients (22). BAFF inhibited IL-10 and TGF-β cytokine secretion, and induced CD4+CD25+ Tregs cell apoptosis in PBC patients (23). Bezafibrate induced BAFF activated B cells, further inhibited BAFF-induced Treg cell apoptosis (23). In an Mdr2 ^−/−^ mice model of PBC, anti-BAFF mAb (SANDY-2) treatment reshaped hepatic B-cell receptor (BCR) repertoire and reduced the titer of the autoantibody antinuclear antibody (ANA) and the levels of its immune complexes. However, targeting BAFF alone could not alleviate hepatic fibrosis (24). Report from a case series found that targeting BAFF with a BAFF receptor inhibitor (belimumab) did not benefit patients with PBC; it could normalize the IgM levels but not alleviate the liver inflammation in patients with PBC (25). Binding of BAFF to its receptor on B cells is critical for the development of splenic transitional B cells to follicular cells and memory B cells. Furthermore, there is a positive correlation between serum BAFF levels, AMA titer and frequency of circulating plasmablasts (26). Hence, the use of anti-BAFF, which is most effective on transitional B cells, could down-regulate the development of B cells to memory B cells. However, BAFF receptor is not expressed on short-lived plasma cells whereas anti-CD20 therapy is effective in depleting peripheral short-lived autoreactive plasma cells. We believe strategies aiming at both peripheral short-lived autoreactive plasma cells and transitional B cells simultaneously could be effective in downregulating the B cell mediated autoimmunity in PBC. Interestingly, a recent study demonstrated that combination of anti-BAFF and anti-CD20 reduced B cells, liver portal infiltration and bile duct lesion in the ARE-Del mouse model of PBC (27). These data shed light on the potential of using a combination of biologics directed at specific immune checkpoints in B cells to treat PBC.

#### 2.1.2 Targeting T cells and related cytokines

The relevance of IL-12/IL-23-mediated Th1/Th17 signaling pathway in the etiopathogenesis of PBC has important therapeutic implications. Livers of PBC patients are heavily infiltrated with T cells. The significance of IFN-γ secreting Th1 T cells in the immunopathology of PBC is well established (28–30). IFN-γ plays a critical role in both AMA production and autoimmune cholangitis (31–33). IFN-γ regulates key signaling pathways such as STAT, p38/MAPK, ERK, and JNK (34), blocking these IFN-γ downstream signaling pathways are likely therapeutic targets in PBC.

The predominant T cell subsets in PBC change as the disease progresses, transitioning from Th1 in the early stages to Th17 in the later stages. Specifically, Th17 activation becomes significantly dominant in the advanced and late stages of PBC. This phenomenon is well demonstrated in livers and peripheral blood of PBC patients (35, 36), as well as in animal models of PBC (37). However, studies directed to monitor and modulate the cytokine profile during disease stages are required to validate the usefulness of this strategy.

Ustekinumab is an anti-IL-12/23 monoclonal antibody used in treatment of several autoimmune conditions. IL-12/23p40 was thought to be a potential target in PBC, via the selective suppression of IL-12 signaling (38). However, IL-12p40 also have been demonstrated to play a vital role as a negative regulator of inflammation in hepatic fibrosis of autoimmune cholangitis; in particular an animal study showed that p40^-/-^IL-2Ra^-/-^ mice expressed more severe portal inflammation and bile duct damage, such as portal hypertension and liver fibrosis (39). A phase 2 multicenter, open-label, proof of concept clinical trial investigating the use of ustekinumab in PBC was disappointing, as none of the patients achieved the primary endpoint. Administration of ustekinumab did not result in a decrease in alkaline phosphatase (ALP) levels of more than 40% in PBC patients who were unresponsive to UDCA treatment (40).

Th17 and mucosal-associated invariant T (MAIT) cells in the liver secreted IL-17 A, which triggered fibrosis via inducing the expression of IL-6 and other pro-fibrotic markers thus suggesting that IL-17A could be a target for anti-fibrotic treatment (41). Both IL-17A and Th17 related cytokines including IL-6 and TGF-β1 participated in the progress of liver cirrhosis. The expression of Th17 associated cytokines was also skewed in patients with PBC. The protein and mRNA levels of IL-1β, IL-6 and IL-23/p19 were up-regulated whereas (transforming growth factor**)** TGF-β1 and FoxP3 expression were down-regulated. Mechanistically, the synergistic activity of IL-17A and TGF-β in the production of IL-6 in dermal and lung fibroblasts depends on the convergent signaling mediated by p38 MAPK, nuclear factor-κB (NF-κB) and PI3K/Akt to some extent. Inhibiting IL-17A negatively affected TGF-β-mediated collagen-I production by SMAD signaling (42). MiR-200c is an anti-fibrotic regulator of cholestatic liver fibrosis. MiR-200c restrained the proliferative and neuroendocrine-like activation of cholangiocytes by targeting Sestrins1 and inhibiting the IL-6/AKT feedback loop to protect against cholestatic liver fibrosis (43). In this line, tocilizumab was found effective and safe in the treatment of rheumatoid arthritis (RA) in patients with PBC (44). However, there is currently only a case report available for this treatment approach, and there have been no clinical trials conducted to investigate its efficacy. Secukinumab is a human monoclonal antibody to IL-17A, which was used to treat psoriasis. Antifibrotic effect was found In 10 psoriatic patients treated with secukinumab, which could improve liver elasticity parameters (45). IL-17i treatment with secukinumab or ixekizumab improved the non-alcoholic fatty liver disease fibrosis score (46). Targeting the IL-17 axis could be a new therapeutic strategy to prevent cirrhosis of PBC. In addition, the anti-TNF-α agents, such as infliximab and adalimumab, potently suppressed IL-12/IL-23 production by inflammatory macrophage by activating FcγRs (47), but anti-TNF-α agent did not play a beneficial effect for development of cirrhosis (48).

In patients with PBC, liver infiltrating CD4+T cells and CD8+ T cells are directed against the lipoic acid binding domain of human the E2 subunits of pyruvate dehydrogenase complex (PDC-E2), and are localized at the pathological biliary epithelial cells (BECs). Elimination of these antigen specific immune responses will be critical in alleviating PBC. A Phase 2a double blind, placebo-controlled study (NCT05104853) using a nano-particle, CNP-104 harboring a PDC-E2 peptide dispersed within a negatively charged polymer matrix of poly (lactic-co-glycolic acid) (PLGA) particle is in progress to evaluate its safety, tolerability, pharmacodynamics, and efficacy in PBC patients unresponsive to UDCA. A recent study demonstrated that peptide-major histocompatibility complex class II (pMHCII)-based nanomedicines displaying PDC-E2 lipoyl domain could reprogramme cognate antigen-experienced CD4+ T-cells into disease-suppressing T-regulatory type 1 (TR1) cells in mice with characteristic pathological features of PBC. Remarkably, recruitment of TR1 cell to the liver leads to restitution of the liver microenvironment, alleviation of autoimmune cholangitis, and reversed established PBC in these mice (49).

Tregs are anti-inflammatory immune cells with a crucial role in the maintenance of peripheral tolerance. The frequency of Treg cells is lower in peripheral blood and livers of PBC patients than in healthy controls. Moreover, the number of *FoxP3*-expressing Tregs was markedly reduced in affected portal tracts in PBC livers when compared with autoimmune hepatitis (AIH) and chronic hepatitis (50). FoxP3 demethylation contributed to the reprogramming of Treg/Th17 phenotype. 5-Aza- 2- deoxycytidine (DAC) can rebuild the balance of Treg/Th17 axis via inhibiting DNA methylation of *FoxP3*, and further alleviate the progression in PBC model. Thus, DAC is also a likely future therapeutic target for reduction of inflammation in PBC (51). PDC-E2 is the major PBC autoantigen and the immunodominant epitope is well-defined at its inner lipoyl domain. The application of chimeric receptor technology to Tregs is a promising approach to induce immune tolerance in autoimmune diseases (52). The humanized mouse model *in vivo* and *in vitro* experiments showed Flagellin-specific human chimeric antigen receptor (CAR) Tregs promoted the establishment of colon-derived epithelial cell monolayers. The potential role of FliC-CAR Tregs in treating inflammatory bowel disease has been documented (53). Development of antigen/liver-specific Treg should be considered for PBC. CAR-Treg can be further gene edited to improve long-lasting outcomes in PBC (54).

AMP-activated protein kinase (AMPK) is a serine/threonine kinase known for its energy sensor function and more recently its ability to maintain *FoxP3* stability and the immunosuppressive functions of Tregs (55). AMPKα1 is a positive regulator of Tregs suppressive function. It activates AMPK to phosphorylate *FoxP3* and regulates its stability. Interestingly, Treg cell-specific AMPK α1 deletion in mice led to compromised Treg cell functions, autoantibodies production, vigorous T cell responses and autoimmune mediated liver injury (56), suggesting that AMPK activation is important for the maintenance of Treg function and the prevention of autoimmune liver disease. Moreover, the study also reported that decreased AMPK phosphorylation in Tregs and reduced in number of Tregs were also evident in PBC patients. Metformin, a pharmacological activator of AMP effectively attenuated the development of experimental autoimmune encephalomyelitis and suppressed systemic autoimmunity in C57BL/6 mice (57, 58). We contemplate that the modulation of AMPK activation treating PBC warrants to be examined.

Imbalance of Tfh cells and Tfr cells has been suggested as one of the underlying factors triggering autoimmunity (59–61). Examination of Tfh cells and Tfr cells in PBC showed that the frequency of circulating Tfh cells is increased whereas the frequency of Tfr cells are decreased in PBC livers when compared with healthy controls. Tfr/Tfh ratio negatively correlated with serum IgM levels. A lower Tfr/Tfh ratio was more prominent in patients with cirrhosis and UDCA non-responders indicating the importance of Tfh and Tfr in the disease development of PBC to UDCA responders indicating the importance of Tfh and Tfr in the disease development of PBC (62). Moreover, cytotoxic T-lymphocyte-associated protein (CTLA)-4 expression in Tfr cells was diminished in PBC. This type of Tfr cells regulated B cell response through CTLA-4 within the germinal center (62). In addition, effector memory CCR7^lo^PD-1 ^hi^ Tfh cells and CCR7^lo^PD-1 ^hi^ Tfr cells were significantly increased in PBC patients, with their levels positively correlated with serum levels of IL-21 and ALP (62). Although UDCA therapy can alleviate such Tfr/Tfh ratio, therapeutics designed for modulating Tfh and Tfr subsets at early disease stages are desired.

Cysteine-rich angiogenic inducer 61 (Cyr61) is an immunoregulatory protein that can modulate the migration of immune cells and promote tissue repair by binding to intergins. Cheng et al. showed that administration of Cyr61 by adenovirus significantly reduced portal inflammation and biliary damage by inhibiting CD8+ T cell cytotoxicity in two mouse models of PBC (63). However, its clinical relevance remains to be determined.

The levels of serum IL-2 involved in liver inflammation and immune process; serum IL-2 levels decreased in PBC. The combination of lower serum IL-2 and higher Total BIL predicted a worse prognosis and higher tendency of liver failure in PBC patients (64). Low dose IL-2 restored immune balance of Sjögren’s syndrome (SS), which was effective and well tolerated in clinical trial (65, 66). Since both pSS and PBC are autoimmune epithelitis it is tempting to speculate that low dose IL-2 could be effective for treating PBC.

#### 2.1.3 Targeting other immune cells and related immune signals

The liver architecture is highly complex with heterogeneous functionally specific cell types such as hepatocytes, cholangiocytes, Kupffer cells, sinusoidal endothelial cells, hepatic stellate cells (HSCs), DCs and immune cells. In addition to T and B cells, immune cell populations such as DCs, NK/NKT cells, monocytes and macrophages are also involved in the pathogenesis PBC (67, 68).

Kupffer cells are sentinels of the liver-specific immune system. When activated, they can produce inflammatory cytokines and eventually damage BECs. Tyrosine-derived Clostridium metabolite p-Cresol sulfate (PCS) effectively reduced PBC related inflammation and regulated Kupffer cell polarization *in vitro* and *in vivo*. Therefore, PCS and its analogues could be effective in treating PBC (69). Mast cell (MC) infiltration are increased during liver inflammation. Activated MCs are source of pro-inflammatory mediators. MCs can indirectly manipulate Tregs functions and inhibit their suppressive and proliferative activity by influencing the intrahepatic microenvironment.

Co-stimulatory signals, cell surface molecules and mediators such as cytokines and chemokines are also vital players in the pathogenesis of PBC. Significant effort in pharmacological design is in progress focusing on those that are pertinent to liver cirrhosis. CTLA-4 gene is the first identified non-MHC susceptibility locus. There is a strong linkage between the CTLA-4 exon 1 polymorphism and PBC (70). Moreover, the number of CTLA-4 copies was found to be positively correlated with inducible co-stimulator (ICOS) and *FoxP3* expressions in PBC patients; lower number of CTLA-4 copies was associated with cirrhosis and decreased expression of CTLA-4 in late stage PBC (71). Lower levels of CTLA-4 mRNA copies were related to the immune suppression caused by cirrhosis. Decreased CTLA-4 and increased ICOS could contribute in the pathogenic process by enhancing B cell and GC response in PBC (62). Preclinical studies on CTLA-4 Ig (abatacept) in PBC murine model showed that treatment with abatacept both before and after immunization improved liver histology, reduced T cell infiltrates and biliary cell damage in the liver. CTLA-4 Ig also inhibited AMA production and autoimmune cholangitis as a preventative agent (72). However, the outcome of abatacept treatment was disappointing in clinical trial; there were no significant changes in serological levels of ALP, ALT, total BIL, albumin, Ig, or liver stiffness from baseline to week 24 after initial treatment (73). The significance of discovering therapies for established PBC cannot be overemphasized, as research studies primarily focusing on “prevention” fail to capture the true clinical conditions that exist in the real world where PBC has already manifested itself. Therefore, it is imperative to shift the focus of research efforts towards finding treatments for PBC that has already developed, to help patients manage the condition effectively.

Programmed death-1 (PD-1), a member of the CD28 superfamily of co-stimulatory molecules, is widely expressed on activated T cells and B cells. Abnormal expression of PD-1 pathway in the liver may contribute to inflammation and autoimmune injury. In the Ae2_a,b_^−/−^ mice model of PBC, PD-L1 expression in mouse BECs was induced by IFN-γ. PD-1/PD-L1 interaction resulted in intrahepatic T-cell activation and the deletion of activated intrahepatic CD8+ T cells in early stage. PD-L1 expression on biliary epithelia can be induced by IL-10 and TGF-β and plays a key role in T-cell tolerance (74). Studies have shown that abnormality in the PD-1 pathway in the liver contributes to inflammation and autoimmune injury in PBC. In patients with PBC, PD-1 was expressed abundantly on liver-infiltrating T cells around injured bile duct (BD) (75),while the mRNA levels of PD-1, PD-L1 and PD-L2 were decreased in the peripheral blood. The PD-1 ligands were regulated by IFN-γ in PBMC of PBC patients (76). Recently, Zhang et al. reported that the expression of PD-1 in peripheral CD8+T cells was decreased, while the level of PD-L1 in human intrahepatic biliary epithelial cell (HiBEC) line was also down-regulated. Hence, silencing of PD-1/PD-L1 pathway with decreased PD-1 expression in CD8+T cells, downregulation of PD-1/PD-L1 in the portal areas, increased CD8+T cell proliferation subsequently enhanced CD8+T cell-mediated cytotoxicity and induced BEC apoptosis (77). Pembrolizumab was the first anti-PD-1 antibody, which produced the therapeutic effects by inhibiting negative signaling via the PD-1/PD-L1 axis, but did not change the phenotype or function of Tregs *in vitro* (78). A case report of a melanoma patient with known PBC/AIH who was administrated with pembrolizumab suggested its safety in humans (79). Further studies including clinical trials are needed to verify the safety and efficacy of PD-1/PD-L1 pathway biologics in reducing biliary damage and liver cirrhosis in PBC.

The trans-membrane protein receptor CD40 and its ligand CD154 (CD40L) are members of the TNF receptor superfamily. CD40 is expressed by a variety of antigen-presenting cells (APCs) and CD154 is mainly expressed on activated CD4+ T-cells, they are synergistically involved in co-stimulation of immune cells. Genome-wide association studies (GWAS) and transcriptome analysis indicated that IFN-γ and CD40L were upstream regulators in both disease susceptibility and activity of PBC (80). Administration of an anti-CD40 ligand monoclonal antibody reduced peripheral T cell activation and improved cholangitis in the dnTGFβRII mice model of PBC (81). In PBC patients, the expression of CD40L mRNA increased while DNA methylation of CD40L promoter was decreased in CD4+T cells, and the level of CD40L and serum IgM were negatively correlated with the CD40L promoter methylation (82). A Phase I/II study (clinicaltrials.gov, NCT 02193360) of an anti-CD40 monoclonal antibody (FFP104; Dacetuzumab/Lucatumumab) in PBC patients was conducted to evaluate its safety, tolerability and pharmacodynamics (83).

All immune cells including T and B cells are derived from hematopoietic stem cells. Mesenchymal stem cells (MSCs) are the most common cell source for stem cell therapy. MSCs played an important role in the modulation of innate and adaptive immune responses and was considered promising therapeutic agents for PBC. MSCs therapy is a potential treatment for PBC. Experimental evidence showed that bone marrow (BM)-MSC might be effective in a PBC mouse model. PBC mouse model induced by injecting polyI:C was treated by allogeneic BM-MSC transplantation, which could regulate systemic immune response and enhance recovery in liver inflammation (84). Human umbilical cord–derived MSCs (UC-MSCs) inhibited the responses mediated by Th1 and Th17, decreased the activities of pro-inflammatory chemokines and alleviated 2-octynoic acid coupled to bovine serum albumin (2OA-BSA)-induced autoimmune cholangitis (85). There have been three MSC based clinical trials for PBC. The first one (NCT01662973) showed that umbilical cord derived MSCs therapy is safe and feasible (86); the second one (NCT01440309) showed that BM-MSC therapy could improve the quality of life and decrease the levels of liver enzymes for as long as 12 months (87), the third one is expected to enroll 140 subjects with 24 month follow up (NCT03668145). MSCs therapy is a promising treatment for PBC, technological advance in generating induced MSCs from differentiation of induced pluripotent stem cells, and application of gene editing and 3-dimensional(3D) culture can enhance the availability and potency of MSCs for therapeutic application (88). We believe that induced MSCs could represent a new breakthrough in therapy for PBC.

### 2.2 Targeting Immune mediators and related signaling pathway

#### 2.2.1 Targeting chemokines of inflammation and fibrosis

Chemokines are signaling proteins that can induce directional chemotaxis in neighboring cells. Hepatocytes, stromal cells and biliary epithelial cells can secrete chemokines mitigating cell migration and tissue infiltration. In PBC, chemokines mediate leukocyte recruitment and subsequent immune mediated damage of intrahepatic BECs. The role of chemokines in pathogenesis of PBC, especially abnormality of C-X-C motif chemokine receptor (CXCR)3 axis, has been reported (89, 90). The levels of chemokines such as IFN-γ-inducible protein-10 (IP-10)/chemokine (C-X-C motif) ligand 10 (CXCL10), monokine induced by IFN-γ (MIG/CXCL9) and CXCR3 were found to be increased in PBC patients and their first-degree relatives, with the expression of IP-10 and MIG in the portal areas. In addition, the frequency of CXCR3-expressing cells in peripheral blood was significantly higher in PBC. CXCR3-positive cells were prominent in the portal areas of diseased livers, primarily on CD4+ T cells (89). The serum levels of MIG and IP-10, CXCR3 expression of peripheral blood mononuclear cells significantly decreased after UDCA administration in PBC patients (90). Serum concentrations of most chemokines primarily responsible for Th1 or Th17 cell chemotaxis, such as IP-10/CXCL10, CXCL11 and fractalkine (FKN)/CX3CL1 were increased throughout the PBC disease course. On the other hand, chemokines predominant for Th2 cell recruitment, for example CCL17, CCL22 and CXCL5, were decreased in PBC patients (91).

The serum level of CX3CL1 was the only chemokine that positively correlates with PBC stage, which increases only in advanced PBC (91). NI-0801 is a fully human monoclonal antibody against CXCL10, which inhibited the combination of CXCL10 with its receptor CXCR3. An open-label, single-arm, phase 2a, proof-of-concept, multicenter study (NCT01430429) was conducted in 29 PBC patients with inadequate response to UDCA. Unfortunately, administration of NI-0801 at a dose of 10 mg/kg did not attain the therapeutic benefit, with headache being the commonly reported adverse event (92).

The level of CXCL13 was higher in serum and liver of treatment-naïve PBC patients. The serum CXCL13 level decreased with oral UDCA, while intrahepatic CXCL13 increased the recruitment of CXCR5+ lymphocytes to liver, eventually resulted in abnormal production of autoantibodies by B cells (93). Studies targeting at intrahepatic CXCL13 should be explored.

In the 2OA-BSA induced PBC model, CCR2-deficient mice manifested milder disease. CCR2 recruited infiltrating Ly6C^hi^ monocytes into the portal zone of livers. Administration of cenicriviroc, a dual CCR2/CCR5 inhibitor, improved liver fibrosis in this PBC animal model (94). Cenicriviroc attenuated disease severity in by decreasing serum bile acids and improving histological severity scores (94). The therapeutic effects of cenicriviroc need to be further investigated in clinical trials.

Substantial data suggested that the FKN–CX3CR1 axis is involved in the pathogenesis of PBC (95, 96), CCL2 and CX3CL1 produced by senescent BECs was up-regulated. These chemokines promoted infiltration of CCR2 and CX3CR1 positive cells and further aggravate inflammation in bile duct lesion in PBC. Anti-FKN mAb E6011 inhibited recruitment immune cells by blocking the FKN–CX3CR1 axis, which was expected to be useful for Crohn’s disease (CD), RA, and PBC (97). A phase II, double-blind, placebo-controlled study showed the clinical benefit of RA patients with inadequate response to methotrexate, although the primary endpoint was not achieved (98). Phase 1 study of E6011 in patients with CD showed it was well-tolerated and might be effective (99). Unfortunately, no clinical trials for PBC have been conducted so far.

#### 2.2.2 Targeting immune signal pathways

Genome-wide studies have identified several candidate genes responsible for antigen presentation and lymphocyte signaling, for example IL-12-JAK/STAT signaling and the NF-κB and TNF signaling pathways (100). To date, studies on signaling pathways in PBC are mainly conducted in animals, with a few clinical trials.

The role of JAK/STAT signaling pathways in many autoimmune diseases has been demonstrated and related drugs were used widely, such as RA (101). Recently the role of JAK/STAT signaling pathway in autoimmune cholangitis was reported. In animal experiments, when ARE-Del+/− mice were treated with the JAK1/2 inhibitor ruxolitinib (102), the level of splenic Tregs increased, and that of splenic CD4+ T, CD8+ T, Tfh cells and germinal center (GC) B cells decreased. The hepatic CD4+ T cells and CD8+ T cells were also suppressed. Ruxolitinib inhibited the expression of IFN-γ gene by the JAK-STAT pathway. A clinical trial for baricitinib (LY3009104) in PBC patients who did not respond to or could not take UDCA (Clinical Trials.gov Identifier: NCT03742973) was conducted to evaluate the efficacy and safety of baricitinib. The study was terminated early because of low enrollment. Two patients were enrolled and completed the trial, one was randomized to receive baricitinib 2 mg/day, and the other received placebo (103). Over the treatment period, a single non-serious treatment-emergent adverse event of moderate sinusitis was reported by the baricitinib treated patient at day 47. This patient demonstrated a rapid and significant decline in ALP, markers of inflammation, pruritus and self-reported depression during a 12-week treatment period, but ALP rebounded to pre-treatment levels during a 4-week post-treatment follow-up. The placebo-treated patient did not show improvement in such biomarkers (103).

With a growing body of evidence identified the important role of the TNF super-family and downstream inflammatory signaling pathways, including NF-κB signaling pathway, in the pathogenesis of PBC, drugs directed at this mechanism is thus of pharmacological interest. The Sirt1 signaling pathway plays a principal role via NF-κB subunit in the development of PBC (104) and therefore a future target for the treatment of PBC. Mammalian Sirtuin-1 (Sirt1), a yeast silent information regulator 2 (Sirt2) homologs, is able to regulate hepatic BAs homeostasis and central metabolic functions through deacetylation. The level of Sirt1 mRNA level was increased in liver tissue of PBC patients, while SIRT1 protein level was up-regulated in the liver during human and murine cholestasis. Over-expression of Sirt1attenuated FXR-mediated inhibition of bile acid synthesis and contributed to the accumulation of bile acids, further induced liver cells apoptosis and aggravated liver inflammation and injury (105). Resveratrol, a Sirt1 activator, suppressed inflammatory responses of PBC by p65 subunit of NF-κB in animal model. Thus far, clinical trials on such related targeted drugs in PBC have not been conducted.

The TLR4/MyD88/NF-κB signaling pathway was activated, and the TLR4 and NF-κB mRNA levels increased in liver tissues of PBC mice. This pathway resulted in liver damage and cell apoptosis by inducing the release of inflammatory factors and producing apoptotic proteins in Poly I:C model mice (106). NF-κB regulated numerous cytokines, and PPARα can interfere with NF-κB signaling. Fenobrate, a peroxisome proliferator activated receptor α-agonist, mediated PPARα activation, regulated inflammatory pathways and inhibited the production of pro-inflammatory cytokines *in vivo* as well as *in vitro* in animal studies. Recently, it was reported that fenofibrate decreased the levels of many pro-inflammatory cytokines by inhibiting nuclear NF-κB p50 and p65 protein expression on the NF-κB signaling pathway, which likely contributed to its anti-inflammatory effects in PBC (107). A CCR2 small interfering RNA silencing (siCcr2)-based therapy by loading multivalent siCcr2 with tetrahedron framework DNA nanostructure (tFNA) vehicle (tFNA-siCcr2) reduced inflammatory mediator production by blocking the NF-κB signaling pathway and attenuated liver fibrosis by regulating the immune cell function in animal experiment (108).

Wnt/β-catenin signaling is critical for various aspects of biliary physiology and pathology, including bile acid secretion, regeneration, and homeostasis. Wnt/β-catenin signaling takes part in hepatocyte–BEC trans differentiation and hepatobiliary repair (109). A crosstalk between TGF-β/Smad3 and Wnt/β-catenin pathway promotes abnormal extracellular matrix production, which is involved in the progression of fibrosis. β-catenin binds to the cofactor CREB binding protein (CBP) or a homolog of CBP P300 and induces target gene transcription. Inhibition of WNT/β-catenin signaling can attenuate fibrosis. Wnt/β-catenin signaling also regulate T cell development and function (110). OP-724 is the specific CBP–β- catenin antagonist. Studies in animal models have verified that OP-724 decreased the Bile acids (BAs) by Egr-1 signaling and exerted anti-fibrotic effects by inhibiting the infiltration of inflammatory cells (111). An open-label phase 1 trial showed in patients with advanced PBC, intravenous OP-724 infusion was well tolerated. Although it did not significantly improve liver function, its anti-fibrotic effects were indicated by decreased in collagen in livers of PBC patients with advanced fibrosis (112).

The Notch signaling pathway was abnormally activated in fibrotic patients (113). This pathway takes part in cholangiocytes proliferation cycle. Inhibition of Notch signaling pathway can prevent biliary liver fibrosis and the abnormal proliferation of cholangiocytes (114). Niclosamide is an FDA approved oral anthelmintic drug. It was found that niclosamide inhibited several intracellular signaling pathways including the Notch pathway during other disease therapy. Niclosamide is a promising antifibrotic agent, which significantly reduced liver enzymes and reduced inflammation by decreasing TNF-α, IL-6, NF-kB and p-STAT3 in PBC animal model (115).

## 3 Targeting bile acid metabolism

**Figure 2:**
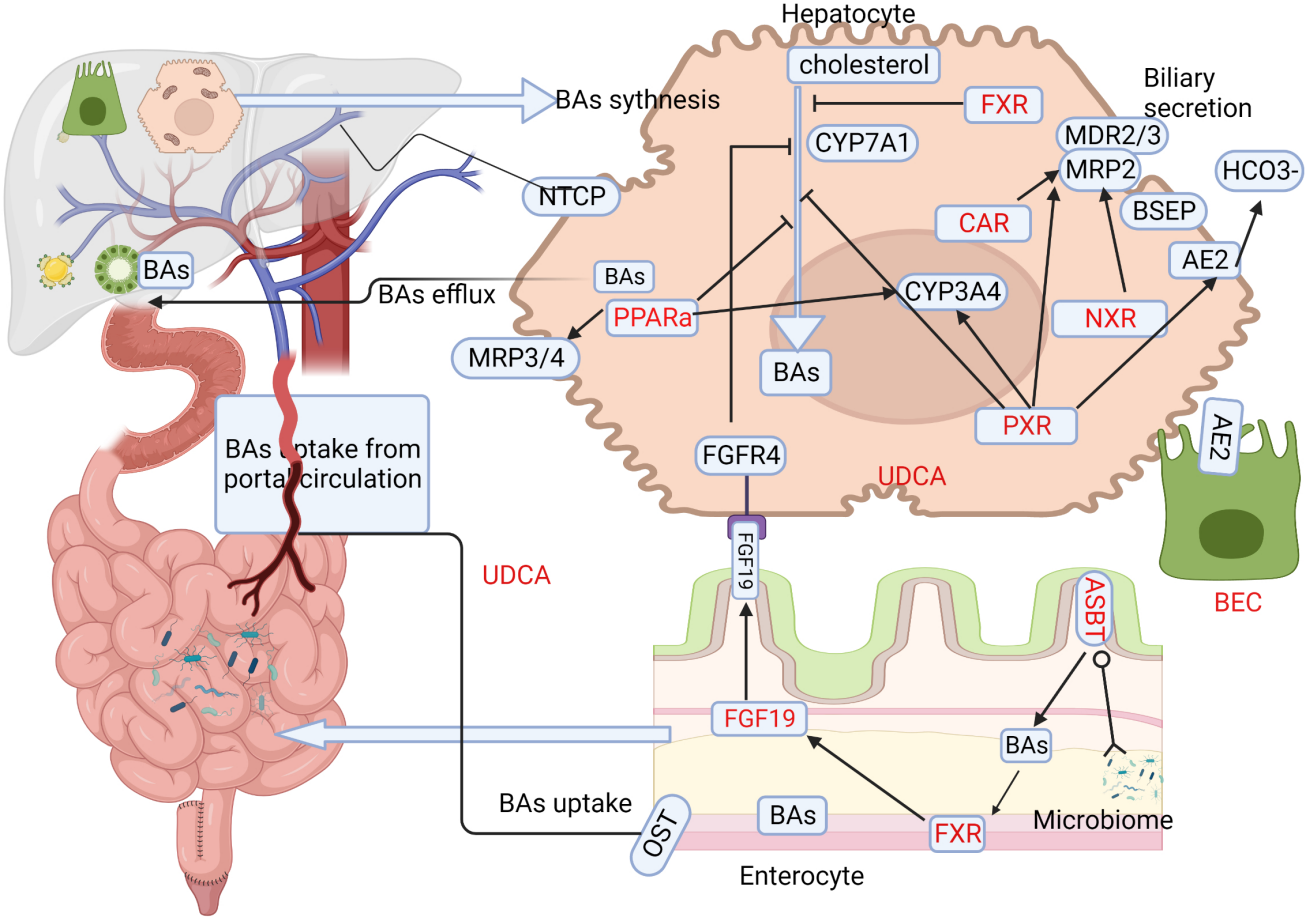
BAs metabolism in liver and intestine and associated therapeutic targets in PBC. The major process of BAs metabolism during synthesis in the liver and their uptake in the enterohepatic circulation provide various windows for developing effective treatments in PBC. Target locations for therapy are highlighted in red. Ursodeoxycholic acid (UDCA) is the classical treatment, and its basic mechanism is to adjust the metabolism of BAs. The first part of PBC treatment involves targeting BAs synthesis, and medication mainly targets nuclear receptors (NRs) such as farnesoid X receptor (FXR), pregnane X receptor (PXR), peroxisome proliferator-activated receptor alpha (PPARα), and constitutive androstane receptor (CAR). Primary BAs are synthesized primarily through the classic pathway, with CYP7A1 being the limiting enzyme. FXR receptors are expressed widely in hepatocytes as well as enterocytes, and BAs inhibit CYP7A1 via the induction of small heterodimer partner in hepatocytes, while in enterocytes, they induce the production of FGF-19, which acts via FGFR4 to inhibit CYP7A1 and BAs synthesis. PXR also plays a vital role in inhibiting CYP7A1. PPARα promotes BAs efflux via MDR3 and MRP3/4, detoxifying BAs and counteracting intrinsic bile toxicity by CYP3A4. The second aspect focuses on the gut-liver axis and gut microbes, including gut microbiota and apical sodium-dependent bile acid transporter (ASBT) inhibitors. Secreted BAs are actively absorbed via luminal ASBT in the distal small bowel, from where they are transported to the portal circulation via organic solute transporter (OST). The reabsorbed BAs are taken up by hepatocyte sinusoidal membrane protein NTCP and re-secreted. The third aspect targets biliary epithelial cells (BECs), as apoptosis of BECs plays an important role in PBC pathogenesis. BECs secrete inflammatory cytokines/chemokines and other antimicrobial molecules, serving as a bridge between bile acid metabolism and the immune response. BAs,Bile acids; PBC, primary biliary cholangitis; UDCA, ursodeoxycholic acid; NRs,nuclear receptors; FXR, farnesoid X receptor; PXR, pregnane X receptor; PPARa, peroxisome proliferator-activated receptor alpha; CAR, constitutive androstane receptor; CYP7A1,cytochrome P450 family 7 subfamily A member 1; FGF-19,fibroblast growth factor 19; FGFR4,FGF receptor 4; MDR3,multidrug resistant protein 3; MRP3/4,multidrug resistance-related protein 3/4; CYP3A4, cytochrome P450 family 3 subfamily A member 4;ASBT,apical sodium–dependent bile acid transporter; OST, organic solute transporter; NTCP, sodium taurocholate cotransporting polypeptide; BECs,biliary epithelial cells.

The metabolism of BAs, especially enterohepatic circulation, plays a vital role in cirrhosis and portal hypertension (116). BAs can activate different receptors, including nuclear receptors (NRs) and membrane receptors and subsequently affect downstream immunological responses. A schematic representation of BAs metabolism in liver and intestine and the BA targets that are of relevance in treating PBC are shown in the figure (**Figure 2**). Autoimmunity and cholangitis have the potential to be improved via regulation of the immune system. BECs survival may be extended by fortifying the bicarbonate umbrella or improving cell membrane integrity (117). Drugs that antagonize BAs toxicity, such as UDCA and nor-UDCA, might be effective at all disease stages. UDCA obtained the cumulative experience over the past decades, but the study aiming at this classical and traditional drug still continue.

### 3.1 The advance and mechanism of classical medicine

UDCA is recommended as the standard first line treatment for PBC. The recommendation highlights a dose–response relationship and the importance of the 13–15mg/kg dose (118). UDCA is a endogenous bile acid, which plays its therapeutic role by multi-aspect mechanisms, including accelerating bile acid enterohepatic circulation, stabilizing the biliary HCO3^-^ umbrella, anti-apoptosis, and anti-inflammatory (119). A large multicenter study indicated that UDCA therapy improved liver transplant (LT)-free survival in all patients with PBC, regardless of the disease stage and the observed biochemical response (118, 120). Unfortunately, although UDCA monotherapy improved overall LT-free survival, approximately 30-40% patients do not respond favorably to UDCA. A recent study showed that PBC patients benefited more from add-on therapies in which UDCA is combined with glucocorticoids or immunosuppressants (121). Bezafibrate combination with UDCA resulted in better biochemical response and lower predicted mortality or LT need than those treated with UDCA alone (122).

### 3.2 Targeting bile acid receptor

BAs consist of a group of multitudes endogenous signaling molecules, that each activates specific receptors such as farnesoid X receptor (FXR), pregnane X receptor (PXR), peroxisome proliferator-activated receptor alpha (PPARα), constitutive androstane receptor (CAR), and vitamin D3 receptor (VDR), as well as the membrane G protein-coupled receptors Takeda G protein receptor 5 (TGR5) and sphingosine-1-phosphate receptor 2 (S1PR2) in the gastrointestinal tract. Physiologically, BAs receptors function as a guard in maintaining gut barrier function and portal pressure. BAs act on a myriad of NRs including FXR and PPAR to regulate cholestasis, inflammation, and fibrosis. It is not surprising that BA receptors are enthusiastically pursued as potential therapeutic targets in PBC.

#### 3.2.1 Farnesoid X receptor

FXR-agonists target both the gut and the liver (**Figure 2**). A well-known agonist of FXR is OCA, which plays anti-inflammatory and anti-fibrotic role by targeting the activation of both liver sinusoidal endothelial cells and Kupffer cells. OCA was recommended as a second-line treatment for UDCA non responders. OCA was shown to be effective and safe in a 3-year clinical trial and follow-up study (123). Its efficacy was also evident in about 43% of UDCA non-responders in real-world (124). The recommended dose of OCA is 5-10mg, with incidence of pruritus increased with dose (125). The FDA issued a new warning in 2021that OCA use in PBC patients with advanced cirrhosis should be restricted due to risk of serious liver injury (126). A combination of UDCA and OCA provided satisfactory clinical outcomes for patients inadequately responded to UDCA monotherapy (127), while add-on therapy with OCA and bezafibrate improved the prognostic markers of difficult-to-treat PBC (128). The triple combination therapy of UDCA, OCA and fibrates improved or normalized the biochemical and clinical features of PBC, such as pruritus, but the safety and side effects needed to be evaluated with longer and larger studies (129). New therapies for PBC targeting NRs including FXR and PXR have generated encouraging results. The combination of FXR agonists and PXR agonists might be a potential approach in avoiding cirrhosis, such as combination therapy of OCA and budesonide for PBC (130). Long-term OCA therapy appears to optimize the prognosis of PBC. OCA is a steroidal FXR agonist, which has poor bioavailability and aqueous solubility. Further studies on pharmacological and toxicological features of OCA and its derivatives may help to enhance its efficacy. Another steroidal FXR agonist, EDP-305, suppressed liver injury and fibrosis without promoting ductal proliferation reaction in two murine models with pre-established biliary fibrosis (131). However, a phase II randomized, double-blind, placebo-controlled study (NCT03394924) on EDP-305 in patients with PBC did not achieve the primary end point (132).

Nonsteroidal synthetic FXR agonists are also therapeutically effective in treating cholestasis diseases. For example, Tropifexor (TXR) binds to the FXR ligand-binding domain and regulated FXR target genes in the liver and intestine. TXR increased FGF19 secretion by activating FXR in the ileum and suppressed bile acids synthesis in the liver. TXR inhibited cholestatic liver injury and fibrosis by modulating the gut-liver axis (133). Clinical trials have shown that TXR was generally safe and well tolerated at daily doses of 30–90 ug, which improved cholestatic markers and the hepatocellular injury marker (134). TXR improved primary bile acid diarrhea by prolonging the ascending colonic transit half-time (135), but the similar side effect of pruritis as OCA still existed because of TGR5 activation.

A Phase 2 clinical trial (NCT02943447) of another nonsteroidal FXR agonist, Cilofexor (GS-9674) yield promising results, with 9% of PBC patients reached the target endpoint of ALP less than 1.67 ULN in the 30-mg group and 14% in 100-mg group. However, 7% of patients in the 100-mg group discontinued treatment due to pruritus. Experimental study demonstrated that a non-bile acid FXR agonist PX20606 greatly improved portal hypertension in a partial portal vein ligation induced non-cirrhotic hypertension. PX20606 also reduced liver fibrosis and sinusoidal dysfunction in a carbon tetrachloride induced cirrhosis rodent model (136). The effects of PX20606 in cholestasis disease such as PBC remains to be determined.

#### 3.2.2 Peroxisome proliferator-activated receptor agonists

Both PPAR and FXR belong to the nuclear receptor family. PPAR regulated bile formation, inflammation and fibrosis as transcriptional modifiers. The PPAR nuclear receptors have 3 isoforms, PPARα, PPARδ and PPARγ. Bezafibrate and Fenofibrate are two major types of PPARα agonist used in treating PBC. Bezafibrate is considered as the third treatment option for PBC, after UDCA and OCA. A clinical trial of add-on therapy with bezafibrate and UDCA for 24 months reported a higher rate of complete biochemical response and an improvement in liver fibrosis in the combined therapy group than the UDCA monotherapy group (137). Fenofibrate is a more selective PPARα agonist, which significantly improved liver biochemical parameters and alleviated pruritus in PBC (138). Fenofibrate in combination with UDCA therapy improved LT-free survival and histological features, including fibrosis and ductular injury, in advanced PBC or liver cirrhotic patients (139, 140). Pemafibrate, a new selective PPARα modulator, was recommended for treating PBC with dyslipidemia, or for patients with poor response to UDCA monotherapy or bezafibrate plus UDCA combination treatment (141). It is also noted that switching from Fenofibrate or bezafibrate to Pemafibrate reduced adverse effects for patients with incomplete response or some renal disorder (142, 143).

Seladelpar (MBX-8025) is a selective PPARδ agonist. A phase II trial reported that patients received Seladelpar could regain normalized ALP levels after 12 weeks of treatment, but the study was terminated early due to increased aminotransferases in the high dose group (144). A phase III trial (ENHANCE) for Seladelpar found that ALP levels were significantly reduced with mild to moderate adverse events in nearly 45% of patients treated with 10-mg dose (145). With its effectiveness in improving liver biochemistry and symptoms, Seladelpar is likely a future second-line agent for PBC (146).

Elafibranor, a dual PPARα/δ agonist, significantly reduced PBC disease activity markers over 12 weeks in a phase II clinical trial (NCT03124108) (147). A double-blind phase III trial (ELATIVE; NCT04526665), aiming at validating effectiveness and safety of elafibranor (80 mg/day) on cholestasis in PBC, is currently ongoing. Saroglitazar is a novel dual PPAR (a/γ) agonist. Clinical trial of saroglitazar showed promising rapid and sustained improvement of ALP in treated PBC patients (148, 149).

#### 3.2.3 Pregnane X receptor

PXR is involved in regulating the biosynthesis, transport, and metabolism of BAs. It regulates BA synthesis by down-regulating cholesterol 7a-hydroxylase (CYP7A1). PXR also has anti-fibrotic and anti-inflammatory properties. Budesonide is a dual agonist of nuclear glucocorticoid receptor and PXR, and has anti-inflammatory as well as immunosuppressive capabilities. Budesonide is also involved in BAs synthesis, metabolism and transport. Unfortunately, budesonide add-on therapy in non-UDCA responsive PBC patients UDCA was not able to reduce liver pathology (150). Moreover, budesonide is not recommended or cirrhotic patients due to risk of increased portal vein thrombosis (132).

#### 3.2.4 Other anti–cholestatic agents beyond NRs

FGF19 is an endocrine hormone, which have antifibrotic effects through reduction of bile acid synthesis and activation of the oxidative stress response. Aldafermin (NGM282), a non-tumorigenic FGF19 analogue, improved cholestatic liver enzymes levels compared with placebo in a clinical trial (NCT02026401) and well tolerated in PBC patients (151). Future studies should focus on decreasing hepatic decompensation or cirrhosis.

### 3.3 Targeting gut-liver axis and gut microbes

The intricate relationship between the gut–liver axis, liver cirrhosis and portal hypertension have positionally centered targeting the gut–liver immune axis as a prospective treatment strategy in PBC. The gut–liver axis highlights the close anatomical and functional relationship between gut and liver. Gut dysbiosis impaired the intestinal barrier and altered human immunity status, enabling bacterial metabolites to reach to the liver through the portal vein (116). The pattern recognition of microbial molecules by cell surface receptors lead to activation of immune system subsequent proinflammatory responses in the liver. Gut microbial dysbiosis was evident in treatment-naïve PBC and could be partially ameliorated by UDCA (152). Gut microbiota and bacterial translocation play an important role in the pathogenesis of PBC, cirrhosis and its complications of portal hypertension (152, 153). The gut microbiota plays a key role in regulating bile acid metabolism, influence intestinal permeability and portal hypertension through the FXR. At the same time, cirrhosis and portal hypertension can have an effect on the microbiome and increase translocation.

#### 3.3.1 The microbiome-based therapies

The three biomes of the microbiome including the immunobiome, endobiome and xenobiome, interact with the host play important roles in the pathogenesis of cholestatic liver disease (154). Molecular mimicry between bacterial proteins could generate humoral and cellular immune response to break tolerance to PDC-E2 revealed the complex orchestration of microbiome and immunobiome in the pathogenesis of PBC (155). Recent studies reported that in patients with liver fibrosis, both the microbiome composition and bile acid composition are altered, suggesting that the gut-microbiota- bile acid axis is a potential target for in treating liver fibrosis (156). The current advances of gut microbiome-based therapies include antibiotics, probiotics, fecal microbiota transplantation (FMT) and precision microbiome-centered therapies. Although these strategies have been successfully used in treating cholestatic liver and intestinal disorders (157), they have not been examined in PBC. Regulating BAs homeostasis by targeting FXR are still the main treatment strategy aiming at gut microbiome in PBC. Both liver biochemistry values and circulating levels of BAs were improved after administrating of cholestyramine PBC patients, their gut microbiota and the composition of BAs were also altered. The effect of cholestyramine on compositional and functional alterations in gut commensal was also evident (158). Bile Acid -microbiota interaction should be explored in treating PBC.

#### 3.3.2 Apical sodium–dependent bile acid transporter inhibitors

The liver has enormous capacity to regulate cholestasis by reducing uptake systems and BAs synthesis. ASBT inhibitors can increase intestinal bile salts absorption and decrease the BA load, and logically should be considered for treating PBC. Several trials have been conducted on small-molecule ASBT inhibitors in PBC. Most of these trials focused on pruritus symptom of PBC. Linerixibat (GSK2330672), a selective inhibitor of ASBT, may treat cholestatic pruritus in this disease setting. Three trials (NCT05448170) (GLIMMER) have documented that Linerixibat effectively reduced pruritus and total serum BA concentrations compared with placebo and also well tolerated (159–161). However, data on preventing cirrhosis are not available. A phase III trial (NCT04950127) named GLISTEN (Global Linerixibat Itch study of efficacy and safety) is ongoing. This study aims to evaluate the efficacy and safety of Linerixibat in 230 participants with PBC and cholestatic pruritus.

Maralixibat (Lopixibat/LUM001/SHP625) is a selective ASBT inhibitor. In a phase II RTC (NCT01904058) study, there were no significant differences in pruritus reduction, cholestasis and hepatocellular injury markers between maralixibat and placebo groups due to a strong placebo effect.

The secretin (Sct)/secretin receptor (SR) signaling pathway regulates the bicarbonate umbrella and stimulates biliary bicarbonate via cyclic cAMP-mediated opening of the cystic fibrosis transmembrane conductance regulator (CFTR) and activates the anion exchanger protein 2 (AE2), which played a key role in maintaining biliary homeostasis. The serological expression of Sct and SR in hepatobiliary and Sct levels were increased in early-stage PBC patients. SR antagonist (Sec 5–27) reduced bile duct damage and liver fibrosis by inhibiting Sct/SR axis in early-stage PBC (162). Sct regulated biliary proliferation and bicarbonate secretion in cholangiocytes via SR in mouse models and human samples of late-stage PBC. Reduced Sct/SR/CFTR/AE2 axis and anterior grade protein 2 (Agr2)/MUC1 levels were detected in isolated late-stage human PBC cholangiocytes, and they were restored after one week of *in vitro* treatment with Sct. Such reduction in biliary Sct/SR/CFTR/AE2 expression and bile bicarbonate levels lead to liver inflammation and fibrosis in late-stage disease in a PBC mice model. Importantly, ductular reaction and biliary senescence were ameliorated by supplying Sct (163). Both short- and long-term Sct treatment promoted bicarbonate and mucin secretion and hepatic bile acid efflux, thus reducing cholestatic and toxic BAs-associated injury in late-stage PBC mouse models (163). This indicated the expression of Sct/SR signaling can be vary with PBC disease stages. Further understanding on mechanism of differential Sct/SR expression in hepatobiliary cells PBC is necessary for designing new diagnostic and therapeutic approaches for the management of PBC.

### 3.4 Targeting biliary epithelial cells

BECs is the major type of hepatic epithelial cells lining both the intracellular and extracellular bile ducts, forming a biliary tree. BECs expresses MHC class I and class II and are active participants in immune-mediated liver diseases. Immunologically, BECs secrete inflammatory cytokines/chemokines and other antimicrobial molecules after TLR stimulation as innate immune cells, present antigens as APCs, as well as secrete IgA and various antimicrobial peptides into the bile (164). BECs mostly expressed CD58 (lymphocyte function-associated antigen 3), CD80 (B7), and CD95 (Fas) (165). Injured and senescent BECs can also regulate the microenvironment around bile ducts by producing associated chemokines and cytokines, which contribute to the bile duct lesions. In 2-OA-OVA-induced mouse model of autoimmune cholangitis, BEC apoptosis was evident in early stage of autoimmune cholangitis and also associated with altered gut microbiota. The apoptosis of BECs was induced bacterial mediated TLR2 signaling (65). Apoptosis of BEC is considered an initial step in the loss of tolerance in PBC, followed by infiltration of CD4+ and CD8+ T cells and liver injury. A recent study showed that emperipolesis is frequently observed in PBC liver sections; such phenomenon is more prominent in early stage than late stage PBC, was mediated by CD8+ T cells with BEC as the host cells (166). Cysteine-rich angiogenic inducer 61 (Cyr61) is a new type of dual immunomodulatory molecule that can regulate both the innate immunity and adaptive immunity. *In vitro* studies showed that Cyr61 regulated intrahepatic immunity by inhibiting the CD8 T cells cytotoxic effects on BECs and inflammation. Overexpression of Cyr61 *in vivo* could alleviate liver inflammation and BECs injury in a mouse model of PBC (63). Cyr61 can be a potential therapeutic candidate for PBC.

### 3.5 Targeting liver fibrosis

Setanaxib (GKT137831) is a selective inhibitor for nicotinamide adenine dinucleotide phosphate oxidase (NOX) isoform 1 and 4. This inhibitor may slow or reverse cholestatic fibrosis (132). It attenuated liver fibrosis and reactive oxygen species production in the MDR2 knockout mice (167, 168). A large phase 2 trial (NCT05014672) on setanaxib was completed, with a significant decrease in liver stiffness and substantial decreases in cholestasis marker after 24 weeks (132).

Lysyl oxidase-like protein 2 (LOXL2) is a key enzyme in the development of organ fibrosis. LOXL2 was associated with BECs injury. It is over-expressed in liver fibrosis and promoted fibrosis progression. Anti-LOXL2 therapeutic antibody inhibited LOXL2, hence attenuated both parenchymal and biliary fibrosis as well as promoted fibrosis reversal in animal experiment (169). LOXL2 is a promising therapeutic target to treat biliary and non-biliary fibrosis. Results from ongoing clinical trials of LOXL2 mAb Simtuzumab on patients with liver fibrotic disease may open the window for new anti-fibrogenic therapy in PBC.

Setanaxib (GKT137831) is a dual Nicotinamide adenine dinucleotide phosphate (NADPH) oxidase (NOX) 1/4 inhibitor, which exerts anti-inflammatory and antifibrotic effects. GKT137831 attenuated liver fibrosis, decreased hepatocyte apoptosis and reactive species of oxygen production in animal models (170). GKT137831 improved markers of cholestasis and inflammation in PBC. A multicenter phase 2 Study (NCT03226067) was designed to evaluate safety and efficacy of GKT137831 in PBC patients with incomplete response to UDCA. A six-week ad-interim analysis showed there was rapid reduction of GGT and ALP levels dose-dependent way and without side effects (171).

## 4 Emerging strategies

Recent advances in molecular and tissue culture technologies have greatly expanded the scope and potential of developing approaches in the treatment of autoimmune diseases (172–175). Here, we discuss some of these unprecedented opportunities and their potential applications in the development of novel PBC therapies in PBC.

### 4.1 Genetics and environmental factors

Genetics has long been recognized to play an important role in autoimmune disease susceptibility. Geo-epidemiological studies in PBC have provided evidence of familial risk, case control studies and genome wide association studies have identified various HLA and non-HLA alleles that are associated with PBC. However, these alleles are non-PBC specific and most of the identified non-HLA loci were also found to be susceptible genes in other autoimmune diseases and different between study populations (176). Extensive studies have addressed the association of HLA class II alleles with the development of PBC. In particular, the DRB1*08 allele family, with DRB1*0801, DRB1*0803, DRB1*14, and DPB1*0301 as susceptible and DRB1*11, DRB1*13 as protective alleles (177–180). A recent study from Japan identified HLA-DQ alleles, DQB1*06:04 and DQB1*03:01, as disease protective alleles (181). A high prevalence of HLA DRB1*0301–DQB1*0201 haplotype among PBC patients in Sardinia was also reported (182).

GWAS analyses from European countries, North America, Japan, and China have identified HLA alleles that possess strong link with susceptibility to PBC and revealed more than 40 non-HLA alleles contributing to PBC susceptibility (183–193) but they can differ among studies and populations. These alleles primarily belong to genes and pathways involved in antigen presentation and production of IL12 (*IRF5, SOCS1, TNFAIP3, NF-κB*, and *IL-12A*), activation of T cells and IFN- production (*TNFSF15, IL12R, TYK2, STAT4, SOCS1, NF-κB*, and *TNFAIP3*), as well as activation of B cells and production of immunoglobulins (*POU2AF1, SPIB, PRKCB, IKZF3*, and *ARID3A*). The association of these immune pathways with the pathogenesis of PBC provide opportunities for strategic therapeutic designs in personalized medicine. Epidemiological studies on PBC showed that frequent exposure to environmental chemicals such as nail polish, chemicals in tobacco smoke, and hormone replacement therapies are significantly associated with an increased risk of PBC (194). Bacterial infection and xenobiotics have been proposed as candidate environmental factors that may explain tolerance breakdown and production of PBC-specific AMAs (195). Large-scale case–control studies have consistently detected an association of PBC with urinary tract infections caused by *Escherichia coli*, as *E. coli* PDC-E2 is molecularly similar to human PDC-E2, the immunodominant target of AMAs (155). Detailed analysis of AMA activity to the human and *E. coli* PDC-E2 indicated that exposure to *E. coli* could elicit specific antibody to *E*. *coli* PDC-E2 resulting in determinant spreading and the loss of tolerance to the human autoantigen (13). Another bacterium of interest is *Novosphingobium aromaticivorans*, a ubiquitous xenobiotic-metabolizing bacterium that produces lipoylated proteins, which are highly reactive with sera from PBC patients (155). The complexity of interactions between genetics and environmental factors (196, 197) together with the changing geoepidemiology and mortality in PBC further highlight the need of novel approaches in order to understand the immunopathogenic basis of PBC to further advance therapeutic approaches towards personalized medicine (198–200).

### 4.2 Epigenetics

Epigenetics is the study of DNA and related factors modifications that are inheritable and do not involve changes in the DNA sequence (201). Epigenetic information controls cellular heterogeneity and identity since the genomic sequence is identical in all cells of the body (201).There are four types of epigenetic information, namely DNA methylation, post-translational changes of histones, non-coding RNAs, and chromatin organization (lack of data on PBC) (202). DNA methylation involves the addition of a methyl group preferentially involving the nucleotide cytosine in CpG sites, which typically results in gene silencing. Post-translational modifications of histones change the DNA accessibility to transcription factors or enhancers and influence transcription and activate or silence genes. These modifications include acetylation, methylation, phosphorylation, ubiquitylation, and sumoylation (201). On the other hand, non-coding RNAs (ncRNAs) are RNAs that do not code for proteins and include two main classes: small non-coding RNAs (miRNAs) and long non-coding RNAs (lncRNAs) (203, 204). These epigenetic modifications could be therapeutic targets in PBC.

Studies have shown the involvement of epigenetic dysregulation in PBC. One study found a significantly reduced methylation level of the CD40L promoter in CD4+ T cells of PBC patients, which led to higher levels of *CD40L* mRNA expression (82). Additionally, the study found that Immunoglobulin M serum levels were negatively correlated with promoter methylation patterns. Studies on methylation patterns in monozygotic twins discordant for PBC and found regions with different methylation patterns on ChrX, with hypermethylation being the common finding in PBC probands (205, 206). In addition, it was shown that imbalance on Treg/Th17 axis in PBC was likely to be affected by the *FoxP3* hypermethylation. In the same study, it was demonstrated that DAC-mediated *FoxP3* demethylation on PBC mice rebuilt the Treg/Th17 balance, resulting in the alleviation of liver lesions and inflammation (51).

PBC affects women more frequently than men, which hampers the drawing of conclusions about potential sex-dependent epigenetic abnormalities. The post-translational modifications of histones have also been implicated in PBC. For example, T lymphocytes from patients with PBC have higher expression levels of *β-Arrestin 1 (βarr1)* than controls, which is involved in T cell activation and has a pathological role in autoimmunity (207). Recently, valproic acid, a histone deacetylase inhibitor, was shown to have antifibrotic effects in the liver and kidney in the experimental adriamycin-induced nephropathy model (208, 209). Although not tested in PBC model, it warrants further study in this condition.

Dysregulation of specific miRNAs has been observed in PBC, and one study found that miR-506 is upregulated in PBC and can target AE2 mRNA, which may contribute to the breakdown of PBC tolerance (210–212). Intriguingly, *miR-506* is located on the X-chromosome. Conversely, few lncRNAs have been implicated in PBC, but *H19* has been identified as a key player in bile duct ligation-induced cholestatic liver injury and is upregulated in PBC and other cholestatic disorders (213). *H19* has multiple functions, including participation in different signaling pathways and functioning as a miRNA sponge (213). There are not reported *in vivo* models or clinical trials implementing RNA interference (RNAi) or small interfering RNAs (siRNA) therapy for treatment of PBC. Further studies targeting pathogenic RNA-associated molecules are warranted. In summary, knowledge on the epigenetic mechanisms and epigenetic contributors will help to understanding the disease process and outcome in patients with PBC so as to develop targeted directed therapies at different stages of disease.

### 4.3 Single-cell RNA sequencing and spatial transcriptomics

Single-cell RNA sequencing (scRNA-seq) technology and spatial transcriptomic (ST) can not only discover new cell types, but also reveal unique changes in each cell, greatly promoting genomics research. GWAS have reported that the association of multiple genetic loci with PBC susceptibility in various populations (214, 215) but without defining any candidate genes. Single cell sequencing analysis revealed that *ORMDL3*+ cholangiocytes had higher metabolism activity and are also play important immune-regulatory roles via the VEGF signaling pathway in the pathogenesis of PBC (216). Recently, Li et al. reported the identification of DUOX2^+^ ACE2^+^ small cholangiocytes in human and mouse livers by ST. DUOX2^+^ ACE2^+^ cholangiocytes interacted with immune cells in the liver portal areas where CD27^+^ memory B and plasma cells accumulated. Interestingly, it was also noted that: a) the number of DUOX2^+^ ACE2^+^ cholangiocytes decreased with the development and progression of PBC; b) the polymeric immunoglobulin receptor (pIgR) was highly expressed in DUOX2^+^ ACE2^+^ cholangiocytes; c) the expression of serum anti-pIgR autoantibodies was highly increased in both positive and negative AMA-M2 of PBC patients (217). Taken together, DUOX2^+^ ACE2^+^ small cholangiocytes and anti-pIgR autoantibody levels can be further evaluated as potential biomarkers in monitoring therapeutic regimens in patients with PBC. Targeting anti-pIgR autoantibodies is likely a potential therapeutic approach in PBC.

### 4.4 Organoids

Organoid technology has evolved with the use of MSCs, liver organoids can mimic different liver disease and increase the translatability of drugs for pre-clinical therapies. Organoids are three-dimensional structures that mimic the structure and function of organs *in vivo*. They are derived from stem cells and can be used to study diseases and test potential treatments (218). Biliary-like cells can indeed be isolated from human bile and cultured long term as biliary organoids (219). Organoids can be used to generate a model of PBC which could be further used to study the disease and its underlying mechanisms (220–222). Researchers can further use organoids to test potential drugs or therapies for PBC, which can then be translated to clinical trials. Some works have developed successful organoid models for primary sclerosing cholangitis which were able to recapitulate the disease inflammatory immune profile (223).

The opportunity to isolate patient biliary stem cells will allow researchers to screen pipe line drugs, develop personalized treatments and therapies that are tailored to the individual patient. This approach can help identify new drugs or repurpose existing drugs for the treatment of PBC (219). On the other hand, organoids could be used to generate new liver tissue to replace damaged or diseased tissue. This approach could potentially be used to treat end-stage liver disease caused by PBC. Using a cell engraftment in human livers undergoing *ex vivo* normothermic perfusion, Sampaziotis et al. (223) demonstrated that extrahepatic organoids were able to successfully repair human intrahepatic ducts after transplantation. It is intriguing that activation of receptor interacting protein kinase (RIPK)3-dependent necroptosis is a core event in PBC (224) and human cholangiocyte organoids can recapitulate cholangiopathy associated RIPK3-dependent necroptosis signaling pathways *in vitro* (218). The potential application of organoids for the development of new treatments for PBC and other liver diseases is promising.

## 5 Current research gaps and potential future developments

The pathogenesis of PBC involves many factors including immunological abnormality, BAs metabolism, gut macrobiotics, BECs injury, gut-liver axis and fibrotic formation. Although with extensive preclinical studies and clinical trials, there does not seem to be a single drug or a single mechanism that is effective in completely halting disease progression and cirrhosis. With the ultimate objective in stopping disease development early enough to avoid cirrhosis and its complications, combinatorial approaches targeting multiple mechanisms and their relevant players are necessary. Multiple immune factors or BAs metabolism play different roles in different stage of PBC disease. Stem cell therapy and anti-fibrotic therapies are potentially useful for preventing progression of PBC. Liver transplantation is currently still the most effective treatment for PBC patients with end-stage liver disease. Long-term studies are needed to evaluate the effectiveness of current treatments and to identify predictors of disease progression and adverse outcomes. Joint effort between clinicians and wet bench scientist work closely together to take advantage of recent research advances such as epigenetics, transcriptomics and the use of organoids technologies to develop unexplored territories in the therapy of PBC. Last but not least, the impact of PBC on patients’ quality of life and well-being is significant, yet there is limited research on patient-reported outcomes in this condition. Future studies should focus on identifying patient-centered endpoints that reflect the impact of PBC in daily life. Clinically, PBC is heterogeneously presented with stages and clinical manifestations; we do not anticipate that there is one “magic bullet” for all PBC patients. Continuous effort in closing the gaps in deciphering mechanisms underlying the disease progress, identifying novel risk loci and vigorous research in candidate drugs will improve the diagnosis, clinical management and outcomes in patients with PBC.

## Author contributions

YY, LG, MR and PL wrote the main manuscript text and prepared all figures. XH and MR, PL revised and edited the manuscript and figures. LG and PL jointly originated and supervised this work. All authors contributed to the article and approved the submitted version.

## Glossary

**Table:**

PBC	Primary biliary cirrhosis
UDCA	Ursodeoxycholic acid
OCA	Obeticholic acid
FDA	Food and Drug Administration
DCs	Dendritic cells
NK	Natural killer
NKT	Natural killer T
AMA	Antimitochondrial antibodies
Ig	Immunoglobulin
IL-6	Interleukin-6
IL-10	Interleukin-10
IFN-γ	Interferon-γ
TNF-α	Tumor necrosis factor-α
ALP	Alkaline phosphatase
BAFF	B-cell -activating factor
BCR	B-cell receptor
ANA	Antinuclear antibody
MAIT	Mucosal-associated invariant T
TGF-β1	Transforming growth factor-β1
NF-κB	Nuclear factor-κB
RA	Rheumatoid arthritis
BECs	Biliary epithelial cells
PLGA	Poly lactic-co-glycolic acid
PMHCII	Peptide-major histocompatibility complex class II
TR1	Regulatory type 1
PDC-E2	E2 subunit of pyruvate dehydrogenase complex
AIH	Autoimmune hepatitis
CAR	Chimeric antigen receptor
AMPK	AMP-activated protein kinase
CTLA-4	Cytotoxic T-lymphocyte-associated protein-4
Cyr61	Cysteine-rich angiogenic inducer 61
Pss	Primary Sjögren syndrome, HSCs, Hepatic stellate cells
MC	Mast cell
ICOS	Inducible co-stimulator
PD-1	Programmed death-1
BD	Bile duct
BAs	Bile acids
HiBEC	Human intrahepatic biliary epithelial cell
APCs	Antigen-presenting cells
MSCs	Mesenchymal stem cells
BM-MSC	Bone marrow-MSC
UC-MSCs	Umbilical cord–derived MSCs
2OA-BSA	2-Octynoic acid coupled to bovine serum albumin
3D	3-dimensional
CXCR3	C-X-C motif chemokine receptor 3
IP-10	Inducible protein-10
FKN	Fractalkine
GC	Germinal center
Sirt1	Sirtuin-1
NRs	Nuclear receptors
LT	Liver transplant
FXR	Farnesoid X receptor
PXR	Pregnane X receptor
PPARα	Peroxisome proliferator- activated receptor alpha
CAR	Constitutive androstane receptor
VDR	Vitamin D3 receptor;TGR5, Takeda G protein receptor 5
S1PR2	Sphingosine-1-phosphate receptor 2
TXR	Tropifexor
FMT	Fecal microbiota transplantation
CFTR	Cystic fibrosis transmembrane conductance regulator
SR	Secretin receptor
AE2	Anion exchanger protein 2
NOX	Nicotinamide adenine dinucleotide phosphate oxidase
LOXL2	Lysyl oxidase-like protein 2
NADPH	Nicotinamide adenine dinucleotide phosphate. ncRNAs, Non-coding RNAs
miRNAs	Small non-coding RNAs
siRNA	Small interfering RNAs
scRNA-seq	Single-cell RNA sequencing
ST	Spatial transcriptomic
RIPK	Receptor interacting protein kinase
